# Isobavachalcone effectively inhibits the growth of *Candida albicans*

**DOI:** 10.1128/aac.00797-25

**Published:** 2025-12-19

**Authors:** Ping Xie, Wenting Zhou, Jiazi Luo, Yuanyuan Dai, Shixian Yang, Shufang Li, Yanqiang Huang

**Affiliations:** 1 Guangxi Technology Innovation Cooperation Base of Prevention and Control Pathogenic Microbes with Drug Resistance, Youjiang Medical University for Nationalities 74654 , Baise, China; 2 Guangxi Zhuang Autonomous Region Engineering Research Center of Clinical Prevention and Control Technology and Leading Drug for Microorganisms with Drug Resistance in Border Ethnic Areas, Baise, China; 3 The University Key Laboratory of Prevention and Control to Drug-Resistant Microbial Infection in Guangxi, Baise, China; 4 Department of Stomatology, Affiliated Hospital of Youjiang Medical University for Nationalities 74654 , Baise, China; 5 Affiliated Hospital of Youjiang Medical University for Nationalities, Key Laboratory of Biomedical Material Research of Guangxi (Cultivation), Baise, Guangxi, China; University Children's Hospital Münster, Münster, Germany

**Keywords:** isobavachalcone, *C. albicans*, ATP biosynthesis, ADK1, antifungal mechanism

## Abstract

*Candida albicans* (*C. albicans*) is a commensal, drug-resistant opportunistic pathogen, and the eradication of invasive candidiasis represents a significant clinical challenge. This study investigated the inhibitory effect of isobavachalcone (IBC) on *C. albicans* growth and elucidated its mechanism. The antifungal activity of IBC was evaluated using minimum inhibitory concentration 90% (MIC_90_) and minimum fungicidal concentration (MFC), combined with murine vaginitis and oral thrush models to assess *in vivo* efficacy. An MTT assay was used to assess drug safety. Mechanistic investigations included cell membrane/wall damage assessments, virulence factor inhibition, oxidative stress evaluation, ATP metabolism analysis, protein expression profiling, and target identification (including RT-qPCR, inhibitor intervention experiments, and related methodologies). The antifungal potency of IBC against *C. albicans* was demonstrated with a MIC_90_ of 2 µg/mL and an MFC of 8 μg/mL. Against strain S393, IBC exhibited potent efficacy with median effective and effective concentrations of 1.301 µg/mL and 1.449 µg/mL, respectively. *In vivo*, IBC significantly alleviated vulvovaginal candidiasis and oropharyngeal thrush, outperforming fluconazole in therapeutic efficacy and mucosal protection. Mechanistic studies revealed that IBC prevented fungal invasion by inhibiting *C. albicans* adhesion and colonization on mucosal surfaces, mitigated inflammation through suppression of proinflammatory cytokine release, and downregulated the expression of *ADE13*, *TPI1*, and *ADK1* genes, with *ADK1* demonstrating the most significant suppression. Furthermore, IBC specifically bound to ADK1, inhibiting ATP synthesis and disrupting cellular energy metabolism in *C. albicans*. IBC represents a promising antifungal drug that acts by downregulating the *ADE13*, *TPI1*, and *ADK1* genes. Its downregulation of ADK1 leads to impaired ATP synthesis, induced DNA damage, and suppressed fungal proliferation.

## INTRODUCTION

*Candida albicans* (*C. albicans*), a dimorphic fungus capable of switching between yeast and hyphal forms, is a commensal organism colonizing the human gastrointestinal tract, oral cavity, and mucosal surfaces. However, it is also a prevalent opportunistic pathogen, exploiting its morphogenetic plasticity to transition from harmless colonization to invasive infections. This phenotypic switching drives pathogenic progression, leading to clinical manifestations ranging from superficial mucocutaneous candidiasis to life-threatening disseminated disease (1). Clinically, *C. albicans*-associated infections account for approximately 9% of hospital-acquired infections, with candidiasis ranking among the most frequent nosocomial fungal diseases (2).

In recent years, the infection rate and therapeutic challenges posed by *C. albicans* have escalated due to the widespread use of chemoradiotherapy, immunosuppressive agents, and the transmission of immunodeficiency viruses (3). Current antifungal drug classes remain largely limited to polyenes, azoles, and echinocandins (4). The nephrotoxicity of polyene antifungals, such as amphotericin B, restricts their clinical utility (5). Moreover, 5-fluorocytosine, a pyrimidine analog, faces resistance in *C. albicans* due to cytosine permease mutations, while β-(1, 3)-D-glucan synthase subunit (FKS) mutations affect echinocandins. Azoles inhibit fungal ergosterol biosynthesis (6), yet emerging resistance poses a major clinical challenge. Notably, *Candida* species develop resistance to azoles through three primary mechanisms: (1) upregulation of point mutations (CDR1/CDR2 and MDR1) and transcription factors (TAC1 and MRR1) in fungal genes, inducing the formation of drug efflux pumps in the cell wall; (2) mutations or overexpression of the ERG11 gene encoding the target enzyme, which alters its binding site and prevents azole binding; and (3) the development of bypass. metabolic pathways via cellular mutations, establishing new drug-resistant metabolic circuits that maintain functional cell membranes while preventing toxic product accumulation (7). Fluconazole-resistant *C. albicans* strains are now classified by the American Center for Disease Control as urgent threats, causing approximately 46,000 hospital-associated infections annually (8, 9). These challenges underscore the urgent need for novel therapeutics targeting drug-resistant *C. albicans*.

Isobavachalcone (IBC), a chalcone flavonoid isolated from the traditional Chinese herb *Psoralea corylifolia L*. (Fructus Psoraleae), was first extracted from its seeds in 1968 (10) and has since been identified in various plant families, including *Fabaceae*, *Clusiaceae*, *Moraceae*, *Schisandraceae*, and *Apiaceae* (11). As a principal bioactive component of *P. corylifolia*, IBC exhibits broad pharmacological activities, including anticancer, antimicrobial, anti-inflammatory, antioxidant, and neuroprotective effects (12). Studies reveal that IBC induces mitochondrial damage through reactive oxygen species (ROS) accumulation and Akt inhibition (13), triggering mitochondria-dependent apoptotic pathways and exerting cytotoxic effects on HepG2 cells, with mitochondrial dysfunction exacerbating in a dose-dependent manner. Furthermore, IBC demonstrates antifungal activity against *C. albicans* and *Cryptococcus neoformans* by disrupting cell walls/membranes, biofilms, and mitochondrial integrity (14, 15). This study validates the antifungal efficacy of IBC through comprehensive *in vitro* and *in vivo* experiments, elucidates its molecular targets and mechanisms against *C. albicans*, and provides a theoretical foundation for the development of novel antifungal agents.

## MATERIALS AND METHODS

### Recovery and cultivation of strains

A total of seven *C. albicans* strains were used, with six strains (BHKS393, BHKS473, BHKS474, BHKS475, BHKS476, and BHKS477) generously provided by Professor Bi Hongkai of Nanjing Medical University. Additionally, a strain of standard *C. albicans* was purchased from the Guangdong Microbial Culture Collection Center. All *C. albicans* strains were inoculated into Sabouraud dextrose agar and cultured at 37°C for 48 h. Single colonies were subsequently transferred to Sabouraud dextrose broth and cultured on a shaker at 37°C for 8–10 h. The resulting fungal suspension was used for subsequent experiments. *Candida tropicalis*, *Escherichia coli*, *Enterobacter hormaechei*, *Enterococcus faecalis*, *Pseudomonas aeruginosa*, *Staphylococcus haemolyticus*, *Acinetobacter baumannii*, and *Proteus mirabilis* were provided by the Microbial Drug Resistance Prevention and Control Center of Youjiang Medical College for Nationalities.

### Drug sensitivity test

IBC (purchased from Chengdu Refensi) was evaluated for its antifungal activity against *C. albicans* (including laboratory strains) using a 96-well plate method. Following dilution by multiple ratios, a logarithmic fungal suspension (1 × 10² CFU/mL) was inoculated, and the observation result was observed at 37°C for 48 h. The fungal solution concentration was adjusted to 1 × 10⁶ CFU/mL, mixed with different drug concentrations, and the optical density (OD) values at 600 nm were measured after 48 h of incubation at 37°C. The median effective concentration (EC_50_) and effective concentration (EC_90_) were then calculated. Additional experimental procedures for IBC against strains, such as *Escherichia coli*, *Morganella morganii*, *Enterobacter hormaechei*, *Enterococcus faecalis*, *Pseudomonas aeruginosa*, *Staphylococcus haemolyticus*, *Acinetobacter baumannii*, and *Proteus mirabilis*, followed the aforementioned protocol, with slight modifications: bacterial suspension working concentration was set at 1 × 10^5^ CFU/mL, and the co-incubation period lasted for 24 h (drug solution combined with bacterial suspension). All experiments were repeated three times.

### Minimum sterilization concentration

IBC (0.5 mg/mL, equivalent to 500 µg/mL) was added to a 96-well plate, and two-fold serial dilutions were performed through the third well (final concentrations: 16, 8, and 4 µg/mL). After inoculation with the fungal suspension, the plate was incubated at 37°C with shaking. Following drug treatment, the fungal suspension was serially diluted and spread onto Sabouraud dextrose agar. Colonies were counted after 48 h of incubation at 37°C. The minimum fungicidal concentration (MFC) was defined as the drug concentration inhibiting 99.9% of fungal growth. The test was repeated three times.

### Cytotoxicity assay

Cell viability was determined using the MTT assay (MTT reagent, OriLeaf, China) in human gastric mucosal epithelial cells (GES-1, Kaiji Biotechnology Co., Ltd., China) and human renal epithelial cells (NRK-52E, kindly provided by Affiliated Hospital of Youjiang Medical University for Nationalities). Survival rate (%) was calculated as (Mean OD_Sample group_ / Mean OD_Control group_) × 100%. The test was repeated three times.

### Construction of the vaginal infection model and thrush model caused by *C. albicans*

#### Construction of the vaginal infection model of mouse

SPF-grade female C57BL/6J mice (6–8 weeks old, provided by Guangdong Vital River Laboratory Animal Technology Co., Ltd., with animal ethics approval number 2023071101 and SPF animal use license number SCXK (Guangdong) 2022-0063) were randomly divided into a model group and a normal group. Low immune response and pseudo-estrus were induced by intraperitoneal injection of hydrocortisone sodium succinate (225 mg/kg) combined with subcutaneous injection of estradiol benzoate (2 mg/mL) (7, 16). Fungal suspensions in the logarithmic growth phase were inoculated into Sabouraud broth supplemented with 10% fetal bovine serum and incubated at 37°C with shaking for 4 h to induce hyphal formation. A sterile cotton ball saturated with 200 µL of fungal suspension (1 × 10⁷ CFU/mL) was intravaginally placed in mice for 30–40 min, avoiding overflow. The infection procedure was repeated once daily for three consecutive days. Following the final infection, mice were observed for vulvar lesions, and vaginal lavage fluid analysis was performed to confirm the successful establishment of the infection model. Upon confirmation of infection, mice were randomly divided into four groups: the normal control group, drug treatment groups, and PBS group, each consisting of six mice (*n* = 6/group). The treatment groups received intravaginally administered fluconazole (10 mg/kg) and IBC at low and high doses (2.5 mg/kg and 10 mg/kg, respectively) for five days, with vaginal lavage fluid collected every other day. All mice were provided with water containing tetracycline (0.83 mg/mL) throughout the experiment (17). Following euthanasia of mice via cervical dislocation, vaginal tissues were harvested to assess fungal colonization and immunohistochemical markers, which were quantified using Image J analysis.

#### Construction of mouse thrush model

Female SPF-grade C57BL/6J mice (6–8 weeks old) were purchased from Guangdong Vital River Laboratory Animal Technology Co., Ltd., with animal ethics approval number 2023071101 and SPF animal use license number SCXK (Guangdong) 2022-0063. Mice were randomly assigned to a model group and a normal group. The model group received intraperitoneal injection of hydrocortisone sodium succinate (225 mg/kg) to induce immunosuppression, followed by anesthesia and application of a *C. albicans*-soaked cotton ball to the tongue surface for 30 min (18, 19). Infection status was monitored daily. The model mice were further divided into fluconazole (10 mg/kg), low-dose IBC (2.5 mg/kg), and high-dose IBC (10 mg/kg) groups, with topical tongue treatment administered for four days. The normal control group, drug treatment groups, and PBS group each consisted of six mice (*n* = 6/group). Following euthanasia of mice via cervical dislocation, half of the tongue tissue was collected for fungal colonization assessment, and the other half was subjected to immunohistochemical analysis. Results were quantified using Image J software.

### *In vivo* safety assessment for IBC

Twelve 6- to 8-week-old specific pathogen-free (SPF) C57BL/6 mice were divided into a control group and a drug-treated group, with six mice in each group. The control group received no drug administration, while the drug-treated group was orally gavaged with a 10-fold therapeutic dose of IBC (25 mg/mL) once daily, with each mouse receiving 0.2 mL per administration. Body weight changes were recorded daily for seven consecutive days. Mice were sacrificed via cervical dislocation, and their livers, spleens, kidneys, and stomachs were examined for damage to obtain a comprehensive assessment of internal toxicity.

### Scanning electron microscope

The blank control group received no drug treatment. Following incubation with the drugs for 4 or 8 h, the samples were washed three times with PBS, treated with an electron microscope fixing solution for 8 h. The fixing solution was discarded dehydrated step by step with 30–100% ethanol solution (10 min each). Samples were centrifuged, and the supernatant was discarded before freeze-drying.

### Determination of ergosterol

According to an optimized reference method, the concentration of the fungal liquid in the logarithmic growth phase was adjusted to 5 × 10^6^ CFU/mL, and the sample was incubated with different drugs for 24 h at 37°C. The fungal precipitate was collected via centrifugation, washed with PBS, and treated with a mixed solution of saponifier, followed by saponification in a water bath at 80°C for 60 min. Subsequently, the sample was extracted with petroleum ether three times, washed with water, and the upper ether layer removed. The sample was then dried to obtain unsaponifiable fat, dissolved in methanol, filtered using a 0.45 µm filter membrane, and analyzed by High-Performance Liquid Chromatography (HPLC) (20). The test was repeated three times.

### Morphological transformation inhibition test

Ten percent calf serum was added to the fungal suspension in the logarithmic growth phase to induce hyphal formation for 2 h, followed by treatment with different concentrations of IBC. After crystal violet staining, the hyphae were observed under a microscope. Both a blank control group and an amphotericin B positive drug group were incorporated, with quantitative analysis performed using Image J following image capture.

### Adhesion inhibition experiment

A fungal suspension (1 × 10⁶ CFU/mL, 100 µL) was inoculated into a 96-well plate, incubated at 37°C for 4 h, and subsequently subjected to MTT assay. The culture medium and non-adherent fungi were discarded, and the samples were washed three times with PBS. The fungi were then cultured at 37°C with IBC at different concentrations for 24 h, followed by the addition of MTT solution to each well. The samples were incubated at 37°C in a dark environment for 2 h, and the absorbance was measured at 490 nm (21). The test was repeated three times.

### Effect of IBC on oxidative damage of *C. albicans*

ROS was detected using the DCFH-DA kit (Biyuntian, China). After drug treatment, 10 µL of fungal suspension was placed onto a glass slide, and fluorescence was observed under a fluorescence microscope, with quantification performed using Image J. The mitochondrial membrane potential of *C. albicans* was detected using Rhodamine 123 (Biyuntian, China), following the same method as ROS detection.

### Determination of ATP content in fungi

The concentration of *C. albicans* suspension in the logarithmic growth phase was adjusted to 10⁶ CFU/mL, incubated with IBC at 2 × MIC (minimum inhibitory concentration), and a blank control group without drugs was established. The intracellular adenosine triphosphate (ATP) content was detected following the ATP test kit instructions (Beyotime, China) at 1, 2, and 4 h. The test was repeated three times.

### DAPI staining

Nuclear concentration and fracture were analyzed via DAPI staining using a DAPI (4′,6-diamidino-2-phenylindole) dye solution (OriLeaf, China). Cells were harvested, washed, and resuspended in 0.5 mL of PBS buffer. Subsequently, they were incubated with 1 µg/mL DAPI in the dark for 20 min. After further washing and resuspension in PBS, the cells were observed under a fluorescence microscope.

### Drug affinity target protein experiment

The total protein of *C. albicans* was extracted, diluted, and incubated with IBC for 45 min at room temperature. Following digestion with different proportions of pronase, the protein was separated by 10% SDS-PAGE gel electrophoresis. Differential bands were identified, and target bands were excised for mass spectrometry identification. Mass spectrometry analysis was performed by Wuhan Jinkairui Biotechnology Co., Ltd., and the data were collected by using QExactive HF mass spectrometer and UltiMate 3000 RSLCnano liquid chromatograph. A protein-protein interaction network was constructed and analyzed by importing differential genes into the STRING database.

### Effect of IBC on the expression of fungal protein

In the experimental group, the *C. albicans* suspension was co-incubated with isobavachalcone at a concentration of 2 × MIC and fluconazole at 2 × MIC, respectively. Here, the 2 × MIC drug concentration refers to twice the minimum inhibitory concentration (MIC) of either isobavachalcone or fluconazole against *C. albicans*. The mixtures were cultured in a shaker at 37°C for 12 h. Then, the samples were centrifuged to collect the fungal precipitate, which was washed with PBS. After protein extraction, the protein content was detected using Biyuntian’s BCA kit. Finally, 10 µL of the supernatant was analyzed by SDS-PAGE.

### IBC specifically binds to ADK1 protein of *C. albicans*

*C. albicans* cells were pretreated with the ADK1 inhibitor ABT702 (MedMol, China) for 1 h, followed by co-culture with 8 µg/mL IBC. The experimental groups included (1) ABT702 pretreatment + IBC, (2) IBC monotherapy, (3) ABT702 monotherapy, and (4) blank control. After 4 h, adenylate kinase was extracted using a cell lysis buffer containing 10 mg/mL cell wall-lytic enzyme (e.g., lyticase) combined with glass bead disruption. Subsequent steps followed the manufacturer’s protocol for the commercial assay kit (MEIMIAN, China).

### Determination of adenylate kinase content

The determination of adenylate kinase content was performed using the adenylate kinase content determination kit (MEIMIAN, China). The methodology was consistent with that employed in adenylate kinase activity detection.

### Statistical analysis

All data were derived from a minimum of three independent experiments and are presented as mean ± standard deviation. Statistical analyses were conducted using GraphPad Prism (v8.0), with one-way analysis of variance applied for multigroup comparisons. *P*-values < 0.05 were considered statistically significant.

## RESULTS

### *In vitro* antibacterial and antifungal activities of IBC

The assessment of MIC_90_ for IBC against laboratory strains revealed distinct antimicrobial profiles: the MIC_90_ against seven *C. albicans* was 2 µg/mL, and *Candida tropicalis* also exhibited a MIC_90_ of 2 µg/mL. In contrast, *Escherichia coli* and other bacterial strains had MIC_90_ values exceeding 128 µg/mL (Table 1). Notably, the seven strains of *C. albicans* displayed severe resistance to fluconazole, ketoconazole, itraconazole, and 5-fluorocytosine, with their respective MIC_90_ values exceeding 64 µg/mL, 16 µg/mL, 128 µg/mL, and 128 µg/mL, respectively. These results indicate that IBC exerts a significant inhibitory effect on drug-resistant *C. albicans*. Detailed information on *C. albicans* strains is provided in Table S1.

**TABLE 1:** Antibacterial and antifungal activities of IBC

Number	Species	MIC (μg/mL)
1	*Candida albicans*	2
2	*Candida tropicalis*	4
3	*Escherichia coli*	＞128
4	*Enterobacter hormaechei*	＞128
5	*Enterococcus faecalis*	＞128
6	*Pseudomonas aeruginosa*	＞128
7	*Staphylococcus haemolyticus*	＞128
8	*Acinetobacter baumannii*	＞128
9	*Proteus mirabilis*	＞128

### Inhibitory activity of IBC on *C. albicans*

**Fig 1:**
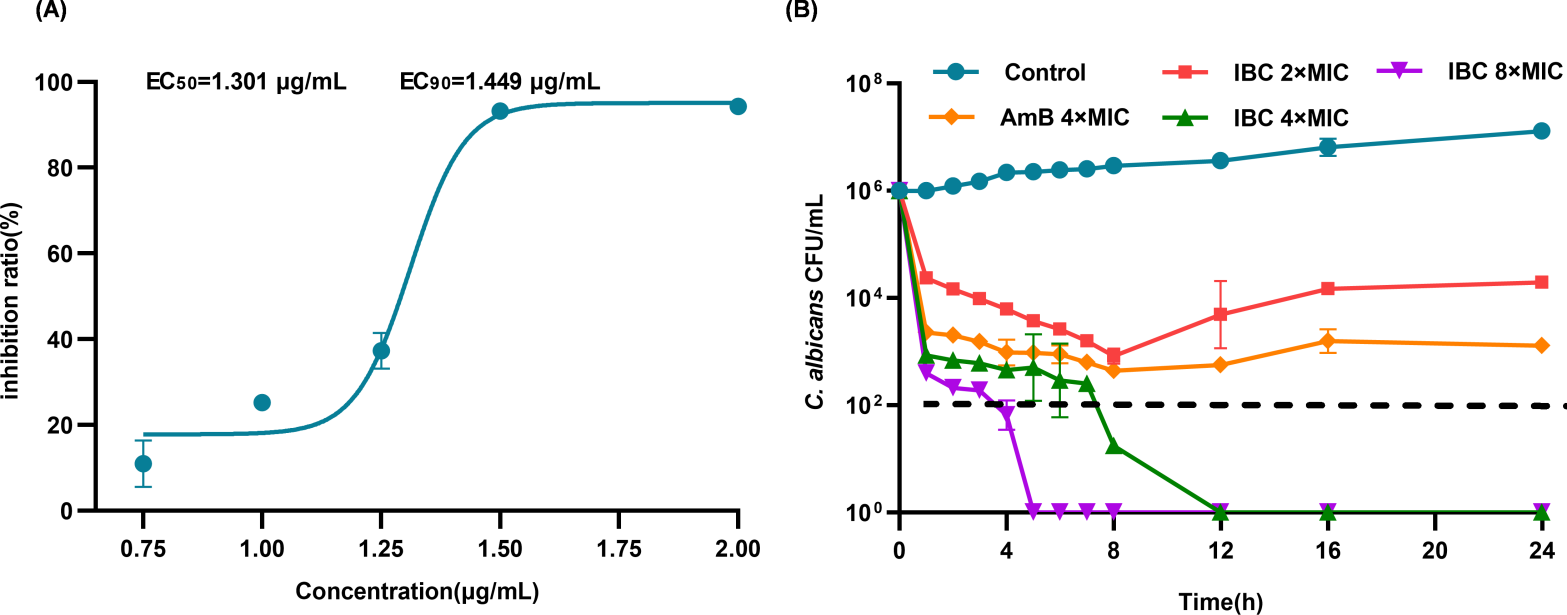
Detection of the antifungal activity of IBC against *C. albicans*. (**A**) Effective concentration (EC)50 and EC90 of IBC against S393. (**B**) Minimum fungicidal concentration of IBC against S393.

Strain BHKS393 exhibited particular susceptibility to IBC, demonstrating EC_50_ (median Effective Concentration) and EC_90_ (Effective Concentration 90) values of 1.301 µg/mL and 1.449 µg/mL (Fig. 1A), respectively. Additionally, the minimum fungicidal concentration (MFC) was determined to be 4 × MIC (8 µg/mL). At this concentration, complete eradication of *C. albicans* was achieved within 12 h. Remarkably, treatment with 8 × MIC (16 µg/mL) significantly shortened the eradication time to 5 h. IBC at 4 × MIC demonstrated superior antifungal activity compared to Amphotericin B at 4 × MIC (Fig. 1B).

### *In vitro* safety evaluation of IBC

**Fig 2:**
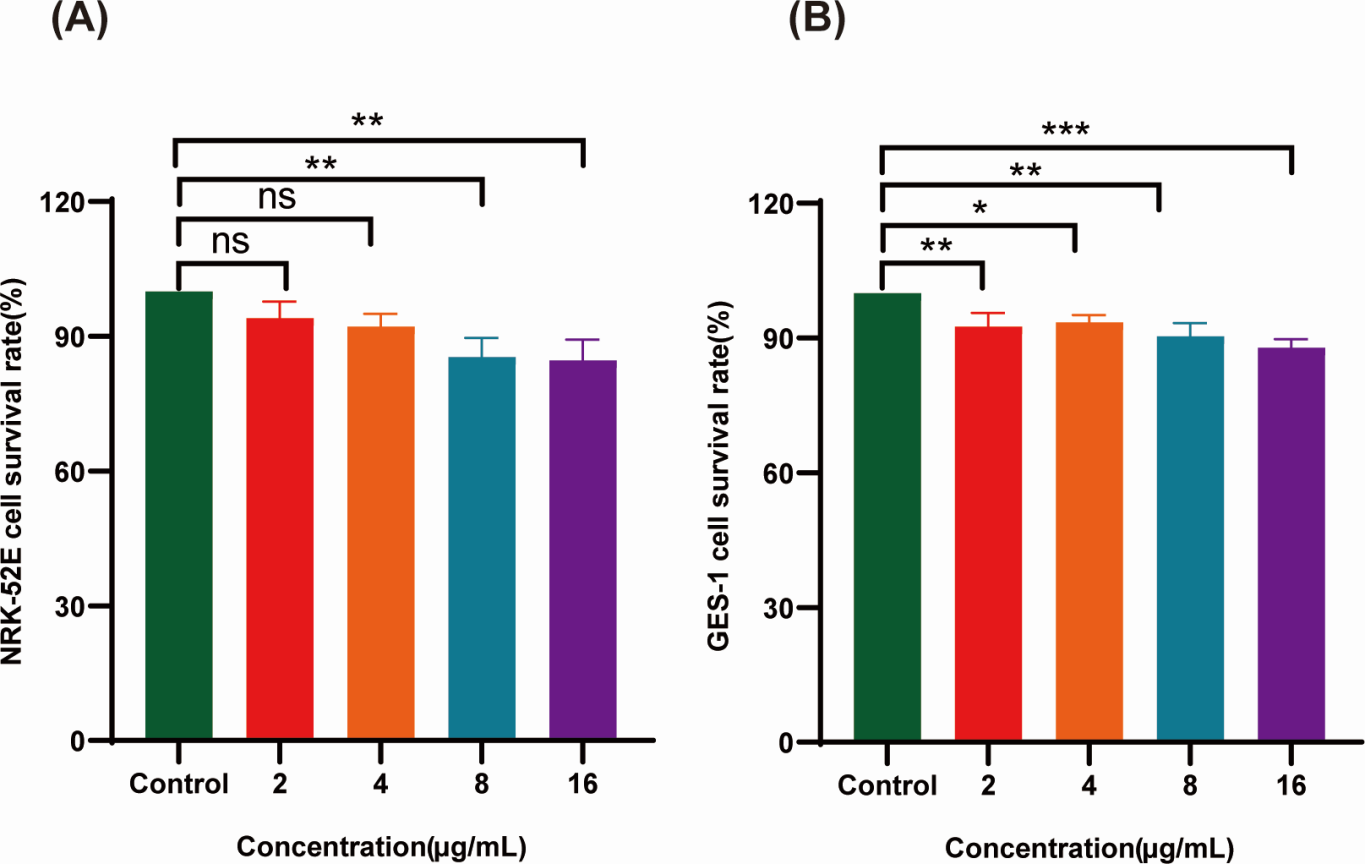
*In vitro* safety evaluation of IBC. (**A**) Survival rate of IBC on human gastric epithelial cells. (**B**) Effect of IBC on the survival rate of human renal epithelial cells. ns, not significant (*P* > 0.05); *P < 0.05; **P < 0.01; ***P < 0.001.

Cytotoxicological assessment via MTT assay revealed minimal cytotoxicity of IBC against normal adrenal epithelial cell lines (NRK-52E) and gastric cell lines (GES-1), maintaining ≥80% viability at 8 × MIC (16 µg/mL) (Fig. 2).

### Effect of IBC on candidal vaginitis

**Fig 3:**
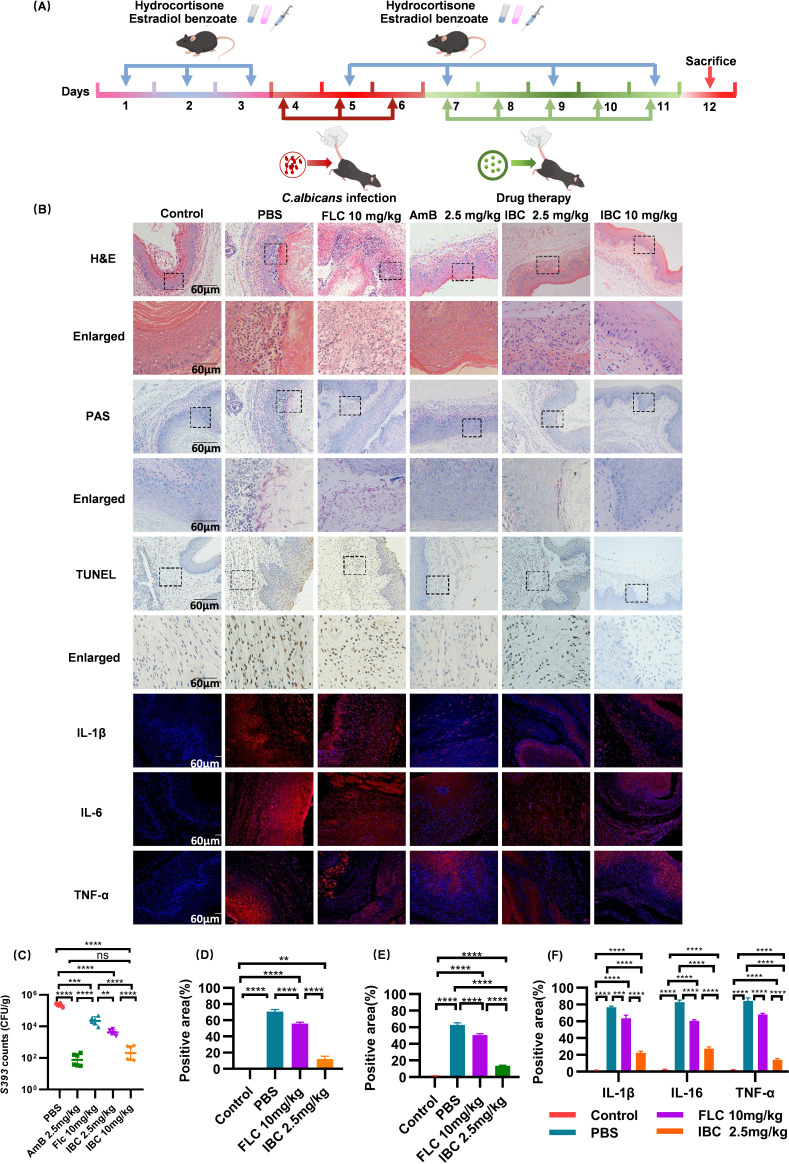
Evaluation of IBC in the treatment of *C. albicans* vaginitis. (**A**) Establishment of animal model. (**B**) H&E staining of vaginal tissue (scale = 60 µm), PAS staining, TUNEL staining, IL-6, TNF-α, and IL-1β immunofluorescence staining. (**C**) Statistics of vaginal fungal load after treatment. (**D**) PAS quantitative graph. (**E**) TUNEL staining of apoptotic cells. (**F**) Immunofluorescence quantification (scale = 60 µm). ns, not significant (*P* > 0.05); **P < 0.01; ***P < 0.001; ****P < 0.0001.

After model building and treatment evaluation (Fig. 3A), H&E and TUNEL staining revealed that the model group had abnormal epidermis with inflammatory infiltration, while the fluconazole group exhibited dermal vacuolation and inflammation. In contrast, the vaginal structure in the high-dose IBC group showed good recovery (Fig. 3B and E). High-dose IBC significantly decreased *C. albicans* vaginal colonization in mice, outperforming fluconazole (Fig. 3C; Fig. S3). PAS staining confirmed inhibition of fungal invasion (Fig. 3D). Fluorescence staining indicated significant downregulation of IL-6, TNF-α, and IL-1β expressions (Fig. 3B and F). These findings suggest that IBC exerts a therapeutic effect by suppressing fungal colonization, alleviating inflammation, and promoting mucosal repair.

### Effect of IBC on thrush caused by *C. albicans*

**Fig 4:**
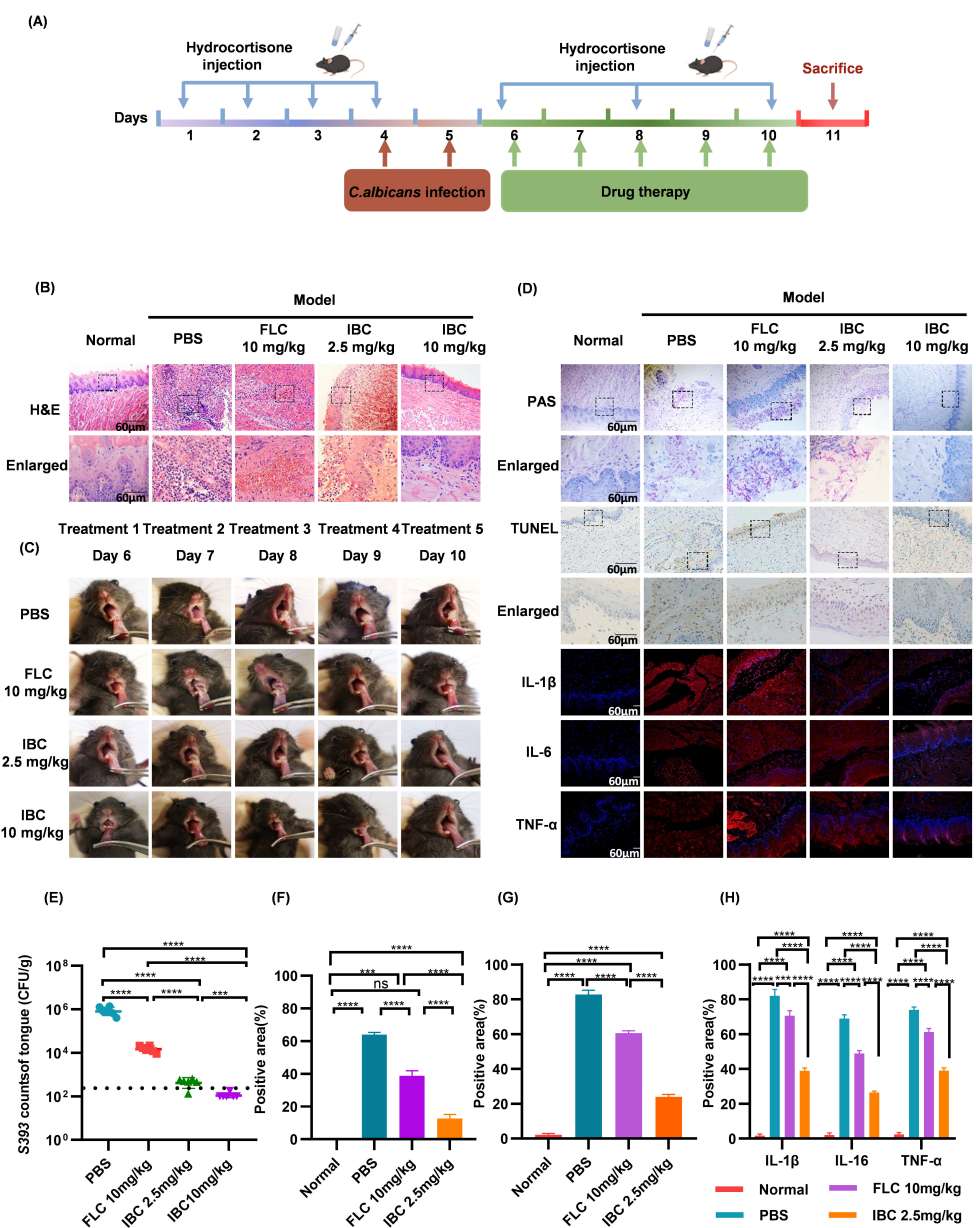
Therapeutic evaluation of IBC in the *C. albicans* thrush model. (**A**) Schematic of experimental thrush model. (**B**) H&E staining of tongue tissue architecture (scale bar = 60 µm). (**C**) Disease progression and therapeutic recovery of lingual lesions. (**D**) Representative PAS staining, TUNEL assay, and inflammatory factor immunofluorescence. (**E**) Microbial load quantification on tongue surfaces. (**F**) PAS staining quantitative analysis. (**G**) TUNEL-positive cell enumeration. (**H**) Inflammatory cytokine (IL-6/TNF-α/IL-1β) quantification. Statistical annotations: ns, not significant (*P* > 0.05); ***P < 0.001; ****P < 0.0001.

In the oral thrush model (Fig. 4A), visual inspection and histopathological analysis revealed distinct morphological differences: specimens from the normal group maintained intact tongue architecture, whereas PBS-treated mice exhibited structural disorganization with inflammatory infiltration. Notably, fluconazole administration caused severe muscular layer deterioration, while the high-dose IBC group displayed well-preserved histological integrity, indicative of potent antifungal protection (Fig. 4B and C). PAS and TUNEL staining quantification confirmed that IBC exhibited superior anti-candidal activity compared to fluconazole, effectively suppressing *C. albicans* colonization (Fig. 4D, F and G). Additionally, IBC demonstrated significant efficacy in reducing lingual fungal colonization (Fig. 4E). Moreover, IBC treatment significantly decreased proinflammatory cytokine levels (IL-6, TNF-α, IL-1β) compared to both PBS and fluconazole controls (Fig. 4D and H), demonstrating dual therapeutic mechanisms through microbial clearance and immunomodulation. These findings collectively indicate the ability of IBC to restore mucosal integrity by simultaneously combating fungal invasion and resolving inflammation-mediated tissue damage.

### *In vivo* safety evaluation of IBC

**Fig 5:**
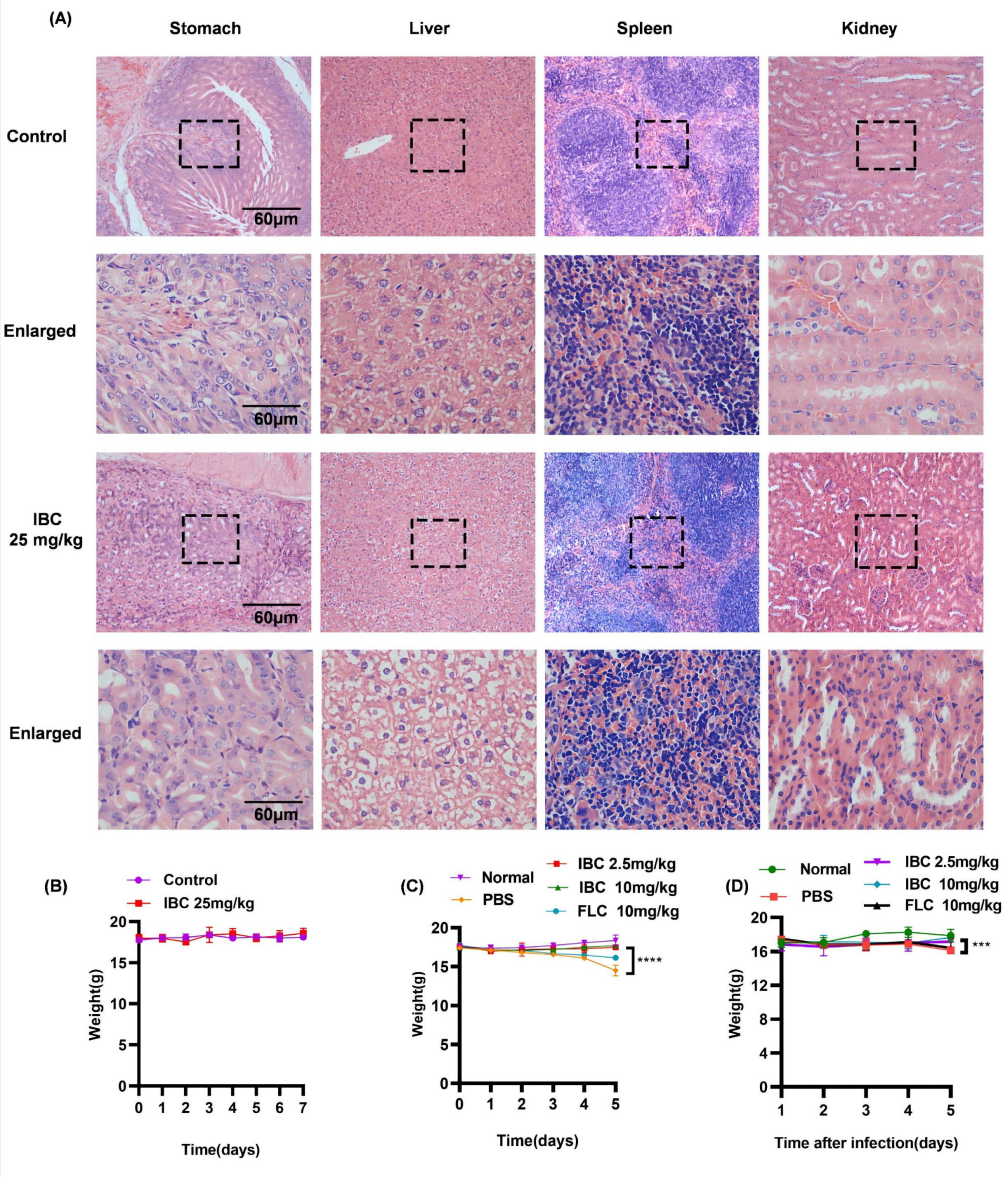
Isobavachalcone *in vivo* safety assessment. (**A**) IBC *in vivo* safety assessment. (**B**) Mice weight curve *in vivo* safety evaluation experiment. (**C**) Weight assessment of oral candidiasis mice treated with IBC. (**D**) Body weight assessment of Candida-induced vaginitis mice treated with IBC. ***P < 0.001; ****P < 0.0001.

Figure 5A demonstrates the *in vivo* safety evaluation experiment of IBC. After one week of gavage administration at a drug concentration ten times the therapeutic dose of IBC, the histological morphology of the stomach, liver, spleen, and kidneys in the drug-treated group appeared identical to that of the normal group. No structural abnormalities or inflammatory injuries were detected, indicating high *in vivo* safety at this concentration.

As shown in Fig. 5B, daily oral gavage for seven days did not result in significant body weight changes in treated mice compared to untreated controls. Treatment-associated weight changes are illustrated for the vaginitis (Fig. 5C) and oral candidiasis (Fig. 5D) models. While a significant difference in body weight was observed between the untreated model group and the IBC-treated group, the latter showed no notable difference compared to the normal group. This suggests that IBC caused no harm to the body during treatment.

### Oxidative damage of IBC to *C. albicans*

**Fig 6:**
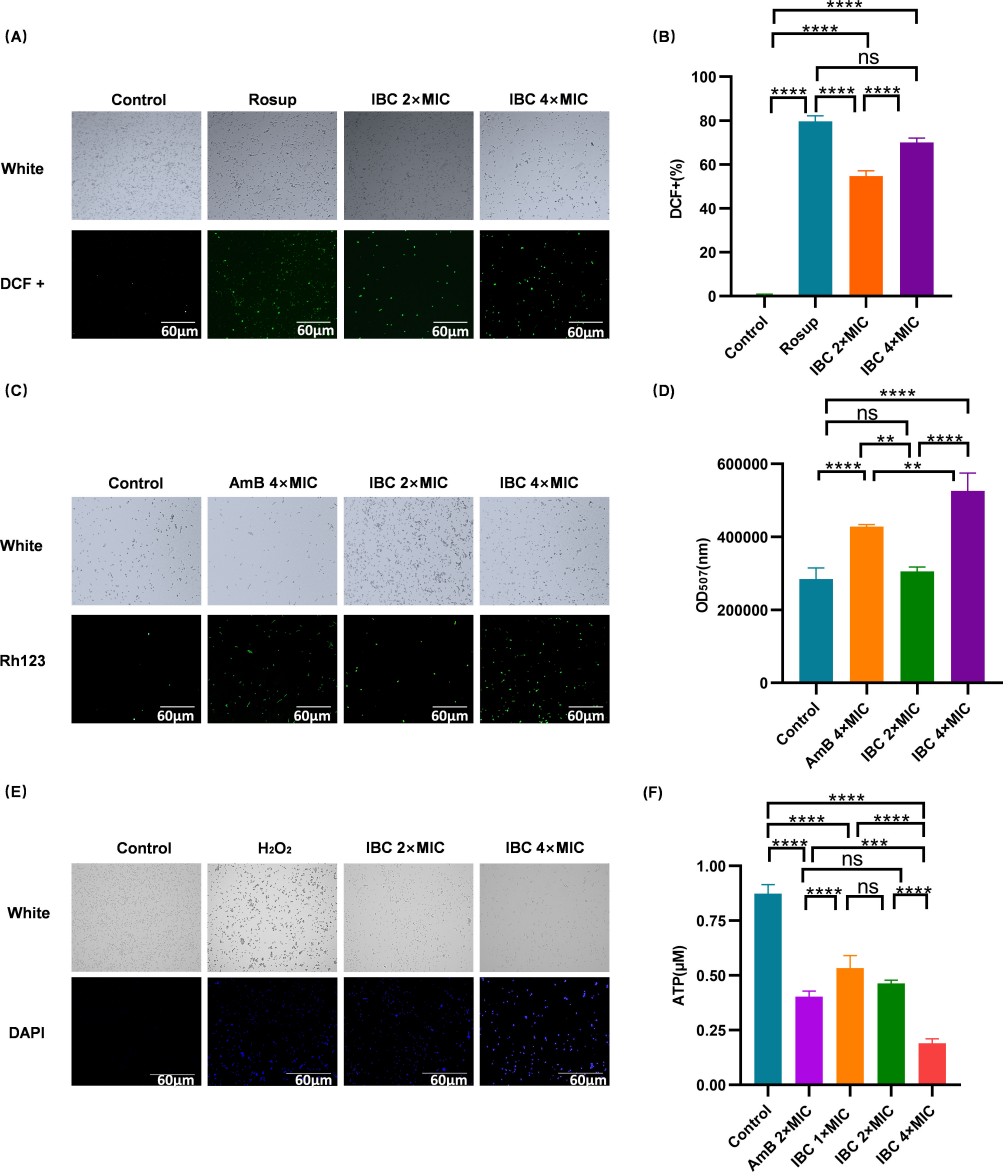
Evaluating the oxidative damage of IBC to *C. albicans*. (**A**) Fluorescence staining to detect the oxidizing effect of IBC on *C. albicans*. (**B**) Quantification of the oxidizing effect of IBC on *C. albicans*. (**C**) Mitochondrial membrane potential detected through Rhodamin123 staining. (**D**) Quantitative representation of IBC-induced mitochondrial membrane potential depolarization in *C. albicans*. (**E**) Labeled DNA-damaged cells through checked ATP. (**F**) Quantification of total ATP content after IBC treatment. ns, not significant (*P* > 0.05); **P < 0.01; ***P < 0.001; ****P < 0.0001.

IBC treatment at 2 × MIC and 4 × MIC induced significant oxidative stress in *C. albicans* within 1 h (Fig. 6A and B). Notably, 4 × MIC IBC led to pronounced mitochondrial depolarization (*P* < 0.0001), surpassing the efficacy of amphotericin B (Fig. 6C and D). Following 2 × MIC exposure, ATP levels exhibited time-dependent depletion, with a 4-hour treatment achieving comparable reductions to amphotericin B at equivalent concentrations (Fig. 6E and F). Notably, DAPI staining revealed dose-responsive apoptosis induction, evidenced by enhanced nuclear fragmentation at elevated IBC concentrations, consistent with the results reported by Wu et al. (22).

### Effect of IBC on the cell structure of *C. albicans*

**Fig 7:**
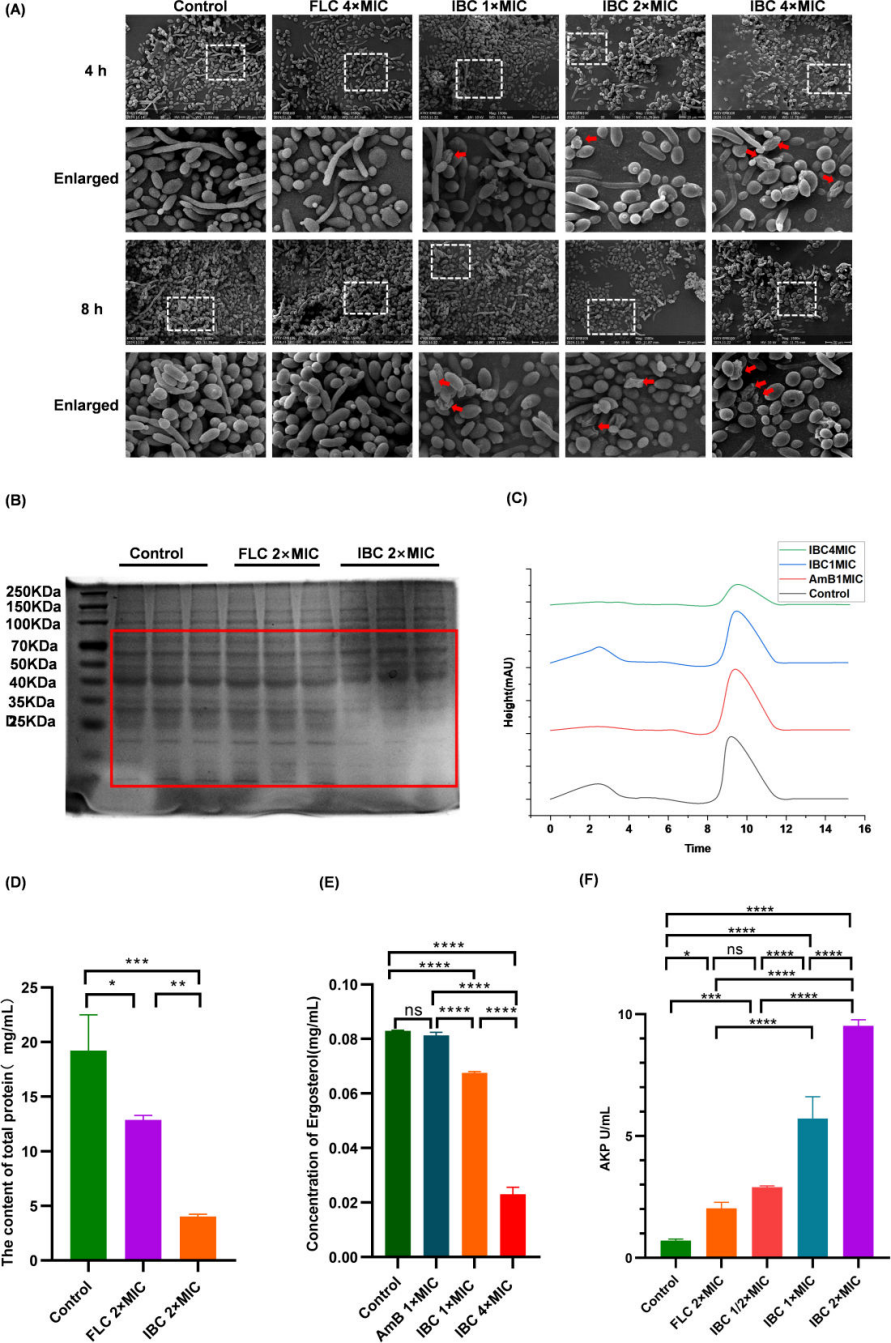
Effect of IBC on the structure of C. albicans S393 cells. (**A**) Cell morphology analysis. (**B**) SDS-PAGE detection of total protein expression. (**C**) Quantification of total protein; (**D, E**). HPLC determination of characteristic peaks and ergosterol content. (**F**) Alkaline phosphatase leakage statistics. ns, not significant (*P* > 0.05); *P < 0.05; **P < 0.01; ***P < 0.001; ****P < 0.0001.

IBC treatment induced cellular distortion and membrane rupture in *C. albicans* strains, as observed via scanning electron microscopy (Fig. 7A). The SDS-PAGE protein bands were absent, and the total protein content of IBC was significantly lower than that of both the control group (*P* < 0.001) and the fluconazole group (*P* < 0.01)(Fig. 7B and C). Figure S1A illustrates that treatment at 2 × MIC caused growth impairment in both SDS (membrane stress) and Congo red (cell wall stress) assays, suggesting concomitant membrane and cell wall damage. The ergosterol content was significantly reduced, with IBC exhibiting a superior concentration-dependent effect on ergosterol content depletion compared to amphotericin B (Fig. 7D and E). Alkaline phosphatase leakage was correlated with higher concentration, indicating progressive cell wall damage (Fig. 7F).

### Inhibitory effect of IBC on virulence factors of *C. albicans*

**Fig 8:**
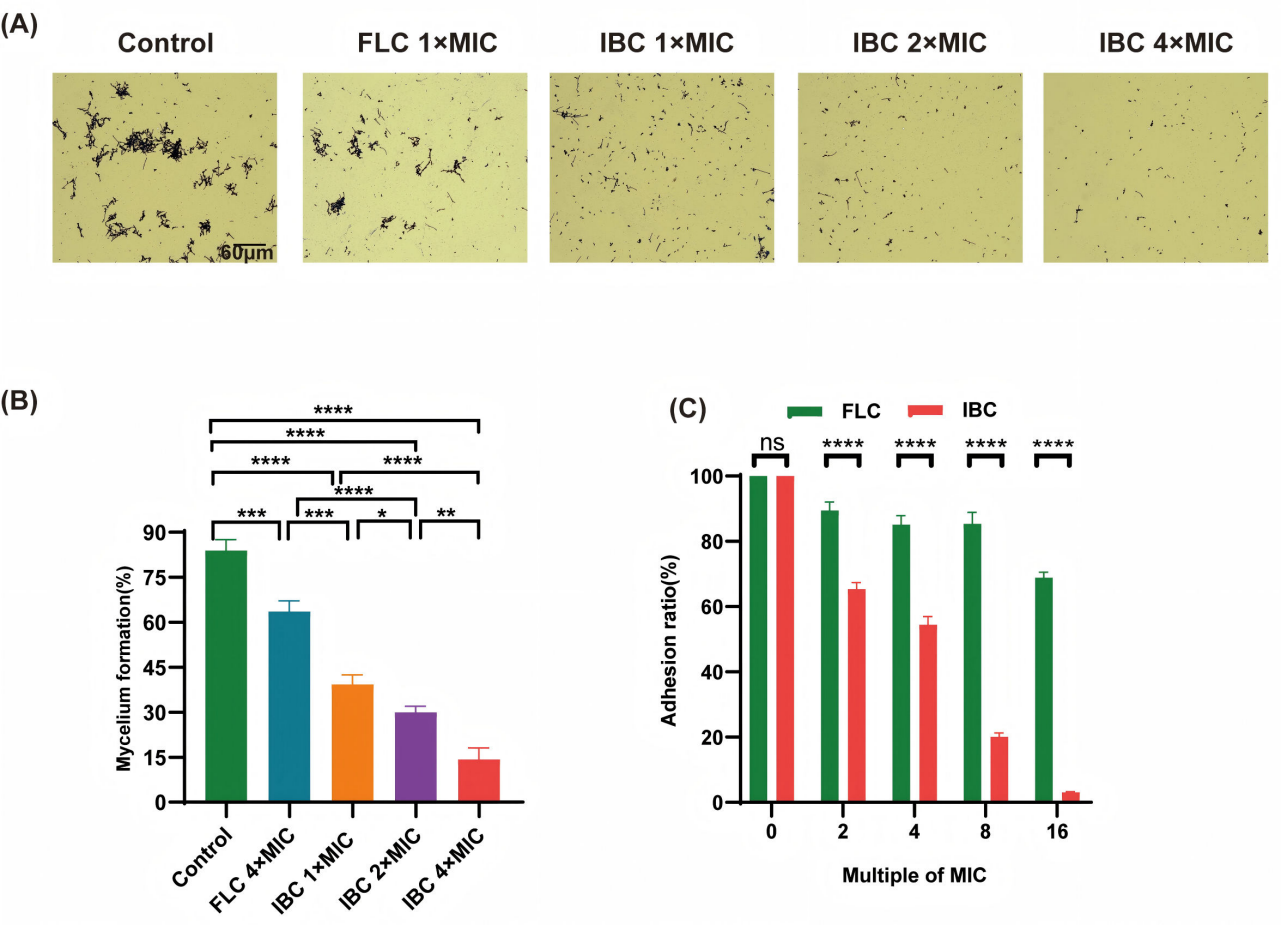
Effect of IBC on the morphological transformation of *C. albicans*. (**A**) Microscopic observation of crystal violet to determine the inhibitory effect of IBC on hypha formation. (**B**) Quantitative graph of the effect of IBC on hypha formation. (**C**) Effect of IBC on C. albicans adhesion inhibition. ns, not significant (*P* > 0.05); *P < 0.05; **P < 0.01; ***P < 0.001; ****P < 0.0001.

*C. albicans* hyphae are more virulent and invasive than spores (23). IBC dose-dependently suppressed mycelium production in *C. albicans*, with a gradual reduction in hyphal growth observed at higher drug concentrations (Fig. 8A and B). This dose-responsive inhibitory effect was further confirmed as shown in Fig. S2A and B. Comparative analysis revealed that IBC exhibited superior inhibitory efficacy at the 1 × MIC concentration compared to fluconazole at its 1 × MIC (*P* < 0.0001). IBC had an inhibitory effect on mature biomembrane, with biofilm survival rates of 62% at 32 × MIC and 84% at 2 × MIC (Fig. S2C). The hydrophobic force of the cell surface affects the adhesion of *C. albicans*. IBC demonstrated a significant inhibitory effect, affecting the adhesion and colonization of *C. albicans*, thereby reducing pathogenicity (Fig. 8C). Furthermore, IBC significantly reduced the hydrophobic force and exopolysaccharides on the cell surface of *C. albicans*, with better efficacy observed with increasing concentration, surpassing fluconazole (*P* < 0.0001) (Fig. S2D and E).

### DARTS-based target profiling identified *C. albicans* proteins mediating IBC’s antifungal activity

**Fig 9:**
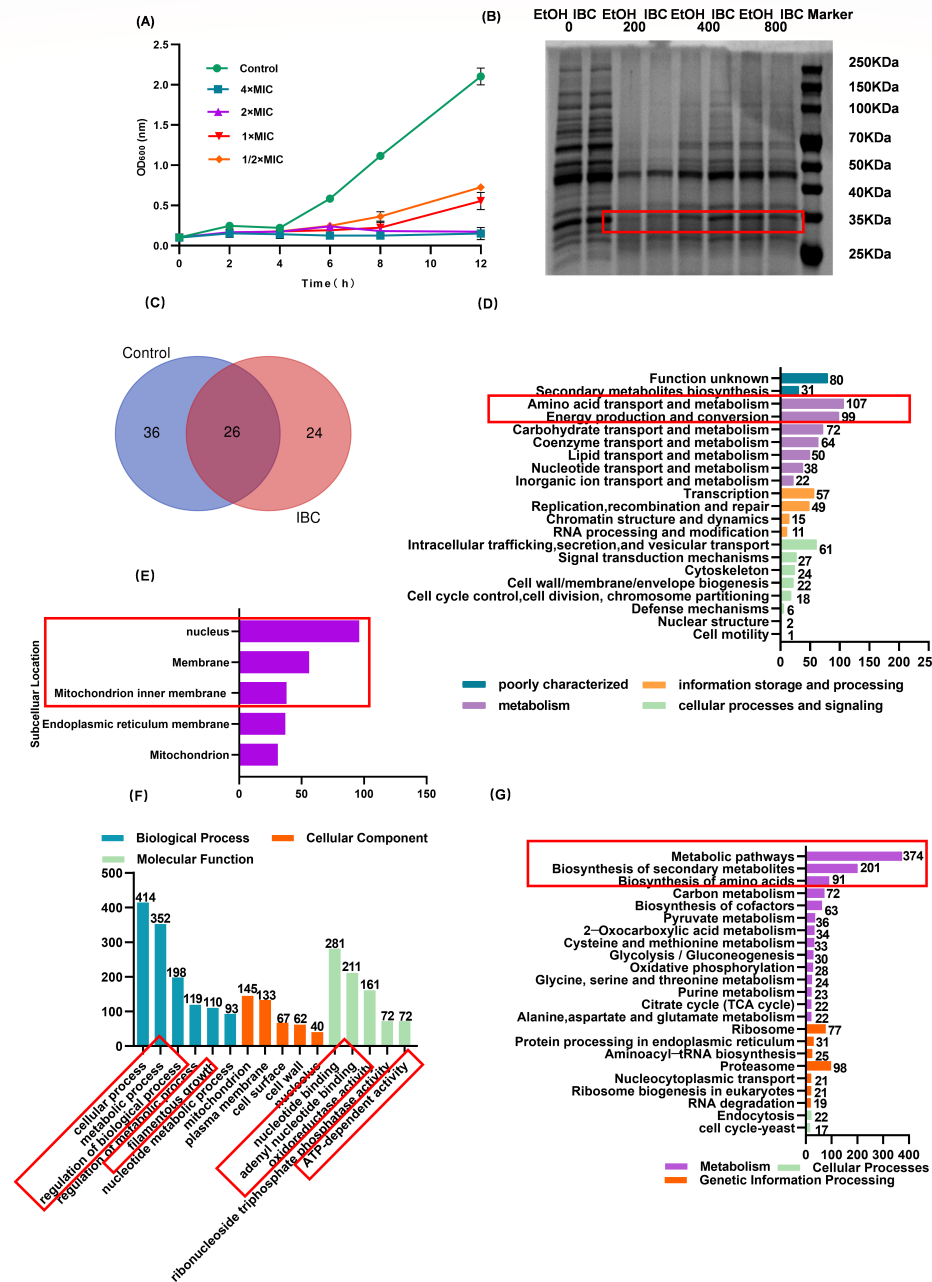
Drug affinity reaction target stability (DARTS) target protein detection. (**A**) Semi-inhibition curve of IBC on *C. albicans* S393. (**B**) DARTS protein screening SDS-PAGE electropherogram with red boxes indicating selected differential bands. (**C**) Venn diagram depicting protein quantity. (**D**) Functional annotation of proteins using COG. (**E**) Subcellular localization prediction. (**F**) GO functional annotation. (**G**) KEGG pathway enrichment.

Drug affinity reaction target stability (DARTS) screening revealed that IBC exhibited a semi-inhibitory effect on *C. albicans* at concentrations of 2 × MIC and 4 × MIC (Fig. 9A). SDS-PAGE analysis identified specific differential protein bands in the 15–25 kDa range within the experimental group (Fig. 9B). The Venn diagram revealed 26 differentially expressed proteins between the control group and the IBC-treated group, with 36 and 24 unique proteins identified in the control and experimental groups, respectively (Fig. 9C). Functional annotations indicated that COG classification primarily involved amino acid transport and metabolism, as well as energy metabolism (Fig. 9D). Subcellular localization prediction revealed that these differential proteins were predominantly distributed in the nucleus, cell membrane, and mitochondrial inner membrane (Fig. 9E). Gene ontology (GO) enrichment analysis showed significant enrichment of differential proteins in molecular functions related to cellular processes, metabolic processes, regulation of metabolic processes, regulation of biological processes, filamentous growth, nucleotide binding, oxidoreductase activity, adenyl nucleotide binding, and ATP-dependent activity. Kyoto Encyclopedia of Genes and Genomes (KEGG) pathway analysis highlighted the involvement of differential proteins in metabolic pathways, biosynthesis of secondary metabolites, and biosynthesis of amino acids (Fig. 9F and G).

### Protein interaction, molecular docking, and RT-qPCR initially confirmed the key gene for the role of IBC

**Fig 10:**
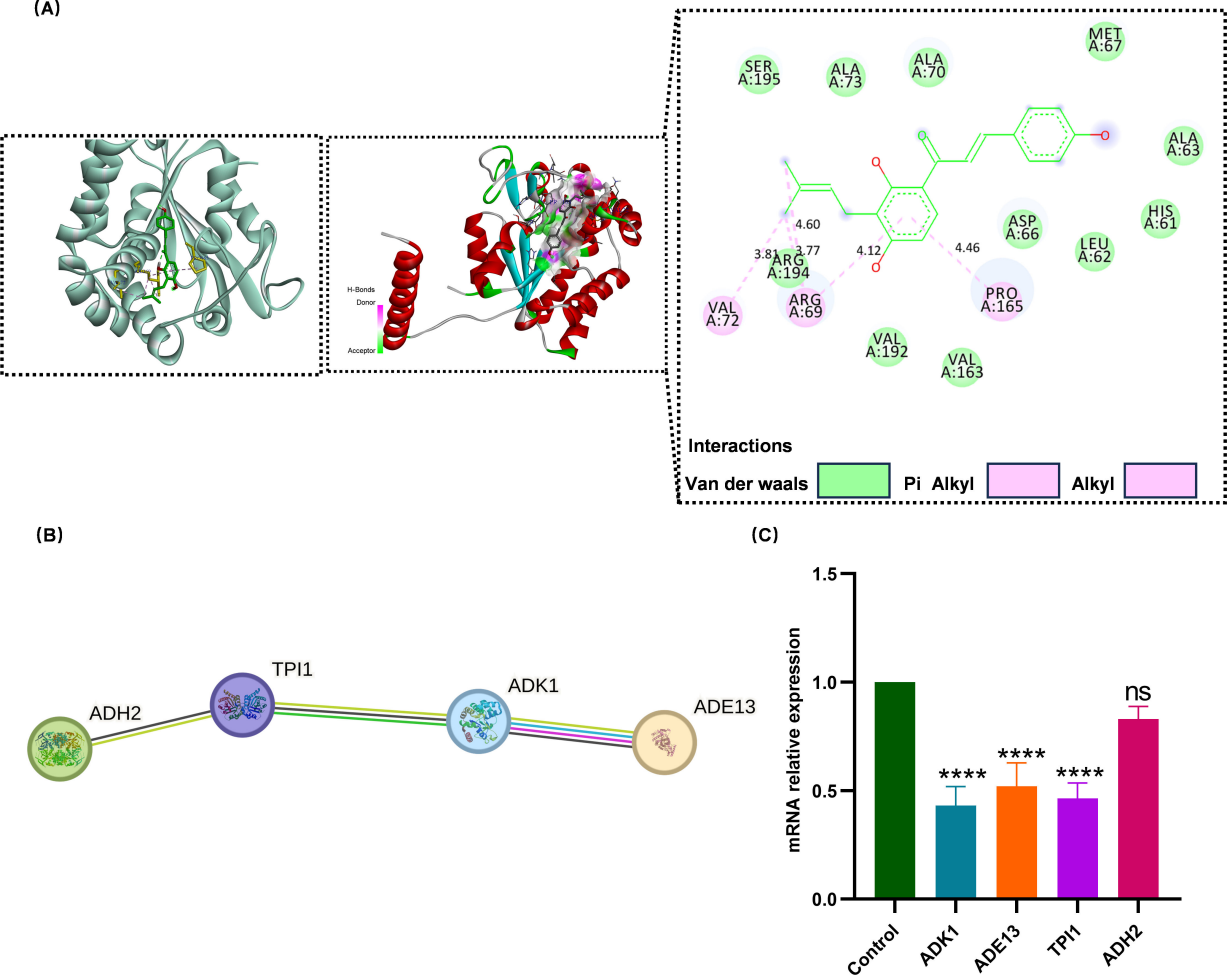
Screening of key genes. (**A**) Molecular docking of ADK1 protein and IBC. (**B**) STRING protein interaction analysis. (**C**) Changes in relative mRNA expression levels of differentially expressed genes in S393 after 8 h of treatment with IBC at 2 × MIC. ns, not significant (*P* > 0.05); ****P < 0.0001.

Integrated analysis of protein characterization, phenotypic assays, and protein-protein interaction network profiling (Fig. 10A) identified four target proteins: ADE13, ADK1, TPI1, and ADH2 (Fig. 10B). All except ADH2 exhibited significant downregulation at the transcriptional level (Fig. 10C). The predicted molecular binding energy of IBC with the three proteins is presented in Table S3.

### IBC specifically binds to the ADK1 protein of *C. albicans*

**Fig 11:**
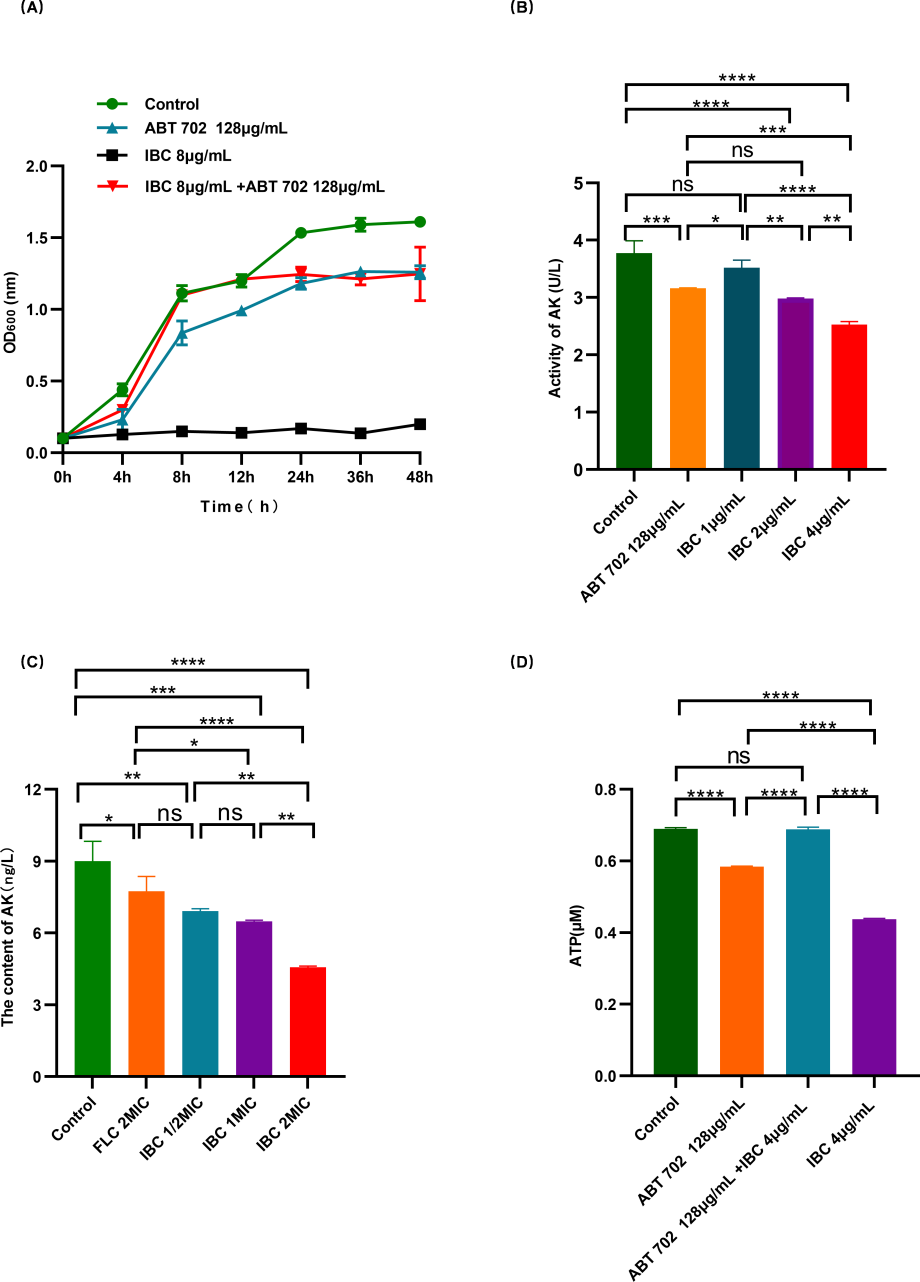
Specific binding of IBC to the ADK1 protein of *C. albicans*. (**A**) Growth curve after IBC treatment on S393. (**B**) Effect of IBC on adenylate kinase activity after IBC treatment on S393. (**C**) Effect of IBC on adenylate kinase content after treatment on S393. (**D**) Effect of inhibitors on IBC’s hinderance of ATP synthesis in *C. albicans*. ns, not significant (*P* > 0.05); *P < 0.05; **P < 0.01; ***P < 0.001; ****P < 0.0001.

Given that ADK1 showed the most significant response to IBC treatment, it was preliminarily explored whether IBC specifically binds to the ADK1 protein of *C. albicans*. As shown in Fig. 11B and C, IBC inhibited both the expression and activity of *C. albicans* ADK1. However, the presence of an ADK1 inhibitor neutralized the antifungal effects of IBC, preventing it from suppressing the growth of *C. albicans* or inhibiting the activity of adenylate kinase (Fig. 11A through C). This suggests that IBC competes with ADK1 inhibitors for binding to the target protein of *C. albicans* ADK1. Similarly, the addition of ADK1 inhibitors prevented IBC from interfering with ATP synthesis in *C. albicans* (Fig. 11D).

## DISCUSSIONS

*C. albicans* causes life-threatening systemic infections (24), with mortality rates exacerbated by diagnostic limitations, therapeutic challenges, and increasing antifungal resistance. The present study demonstrates that IBC exhibits potent anti-*candidal* activity against azole-resistant strains (MIC_90_ = 2 μg/mL; MFC = 8 μg/mL; EC_50_ = 1.301 μg/mL; and EC_90_ = 1.449 μg/mL). Additionally, IBC effectively inhibits clinical isolates of *C. albicans* resistant to fluconazole, ketoconazole, itraconazole, and 5-fluorocytosine, and its inhibitory effect was comparable to that of amphotericin B, offering a promising candidate drug to mitigate *C. albicans* resistance (Table S1). The antifungal mechanism primarily involves the induction of endogenous ROS bursts, resulting in mitochondrial membrane depolarization, DNA fragmentation, and ATP biosynthesis blockade, ultimately leading to organelle damage and apoptosis (25). These findings suggest that IBC possesses therapeutic potential in disrupting fungal cellular homeostasis through oxidative stress pathways.

Excessive ROS generation disrupts fungal macromolecules and organelles, notably inducing membrane lipid peroxidation and structural damage (26). Ergosterol, as a fundamental membrane component, regulates multiple cellular biological functions (27). Our findings demonstrate that IBC effectively suppresses ergosterol biosynthesis and global protein expression in *C. albicans*. Furthermore, ROS overaccumulation indicates a compromised antioxidant defense capacity, which is a critical determinant of fungal virulence traits (28). Through multidimensional virulence factor assays, we confirmed that IBC exhibits potent inhibition of *C. albicans* pathogenicity, likely attributable to compound-induced oxidative damage.

*In vivo* experiments demonstrated the effectiveness of IBC in treating oral thrush and vulvovaginal candidiasis caused by *C. albicans*. The hyphae of *C. albicans* invade host epithelial cells through endocytosis, with the transition from yeast to hyphal form closely linked to the incidence of vulvovaginal candidiasis and oropharyngeal candidiasis, potentially leading to excessive immune responses and mucosal inflammatory damage (29, 30). The current study demonstrated through mouse experiments that IBC can inhibit *C. albicans* invasion into mucosal tissues, promote mucosal recovery, and reduce inflammatory cell infiltration, which is associated with its ability to disrupt hyphal activity.

The mechanisms by which IBC inhibits *C. albicans* were elucidated through DARTS experiments, bioinformatics analysis, and RT-qPCR technology. GO enrichment analysis revealed that IBC significantly affects cellular processes, regulation of biological processes, and metabolic processes, as well as exhibiting actions on nucleotide binding, adenyl nucleotide binding, and ATP-dependent activity. Protein interaction network analysis identified key targets, namely ADE13, ADK1, TPI1, and ADH2. RT-qPCR confirmed that IBC downregulates the first three genes. ADE13 can reduce reactive ROS production in fungal cells, enhance antioxidant enzyme activity, mitigate oxidative damage, and promote the conversion of ATP, ADP, and AMP (31). The induction of ROS production by IBC, which leads to oxidative damage, may be related to the downregulation of ADE13. TPI1 facilitates the binding of *C. albicans* to host extracellular matrix proteins, thereby enhancing its adhesion and colonization capabilities (32). By downregulating TPI1, IBC reinforces its ability to reduce the virulence of *C. albicans*.

ADK1 (adenosine kinase) is a key enzyme in purine metabolism, catalyzing the phosphorylation of adenosine to produce AMP (adenosine monophosphate). It plays a central role in cellular energy metabolism, nucleotide homeostasis, and signal regulation. In *C. albicans*, reduced ADK1 expression or activity may disrupt the cross-interaction between metabolic networks and virulence regulatory pathways, triggering oxidative stress and ATP leakage, inhibiting hyphal formation and biofilm development, and ultimately undermining the fungus’s survival, proliferation, and pathogenic capabilities (33). In this study, ATP assays revealed that IBC significantly reduced ATP synthesis, which may be related to its disruption of ADK1 activity. Moreover, measurements of ADK1 enzyme activity and content demonstrated that IBC not only inhibits ADK1 activity but also reduces its levels. Introduction of an ADK1 inhibitor attenuated the antifungal activity of IBC against *C. albicans*, preventing effective inhibition of fungal proliferation, ADK1 activity, or ATP synthesis. These experiments demonstrate that IBC can specifically bind to the ADK1 protein, thereby blocking its active site. By specifically binding to the ADK1 protein, IBC significantly inhibits its function. As a key mitochondrial enzyme regulating ATP and ADP metabolism, ADK1 displays significantly reduced activity and content in response to IBC treatment, leading to impaired ATP synthesis. These preliminary findings confirm that ADK1 is the core target of IBC against *C. albicans*: inhibition of ADK1 activity results in failure of IBC to effectively control fungal proliferation or ATP metabolism. Mechanistic studies have shown that IBC can exert its effects by blocking the active site of ADK1. However, to confirm this targeted effect further, molecular cloning, protein interaction analysis, and crystallization detection are needed for further verification, which will be the direction of our future research efforts.

### Conclusions

This study confirms that IBC is an effective compound against *C. albicans*, with a MIC_90_ of 2 µg/mL for inhibiting its growth. IBC exerts multifaceted damaging effects on *C. albicans*, which include (1) disrupting the cell wall (2); reducing ergosterol synthesis in the cell membrane (3); attenuating fungal invasive virulence (4); inducing ROS production leading to mitochondrial damage and decreased ATP levels; and (5) causing nuclear DNA damage. The potent antifungal activity of IBC is primarily associated with its downregulation of key metabolic pathway genes in *C. albicans*, namely *ADE13*, *TPI1*, and *ADK1*. Notably, ADK1 is likely the primary target gene of IBC.

## Data Availability

Data supporting this study are retained in the research team’s database. Requests for data access can be sent to the corresponding author via the provided email, with a reasonable research purpose required for review and approval.

## SUPPLEMENTAL MATERIAL

Supplemental material aac.00797-25-s0001.docx Fig. S1 to S4; Tables S1 to S3.

ASM does not own the copyrights to Supplemental Material that may be linked to, or accessed through, an article. The authors have granted ASM a non-exclusive, world-wide license to publish the Supplemental Material files. Please contact the corresponding author directly for reuse.
