# Supplementary material for: Isobavachalcone effectively inhibits the growth of Candida albicans
Source: Antimicrob Agents Chemother. 2025 Dec 19;70(2):e00797-25. doi: 10.1128/aac.00797-25 (PMC12888897; doi:10.1128/aac.00797-25)
Supplement: Supplemental material — Fig. S1 to S4; Tables S1 to S3. [file aac.00797-25-s0001.docx]

Supplementary Material

# Supplementary Figures

1.1 Method

1.1.1 Effect of Isobavachalcone on the Cell Wall of *Candida albicans*

Sodium dodecyl sulfonate (SDS, 0.01% w/v; Yuanye, China) and Congo Red (1 mg/mL; Yuanye, China) were added to Sabouraud Dextrose Agar (SDA) medium, respectively (1). Isobavachalcone was mixed with the fungal suspension in a sterile 96-well plate, and amphotericin B (AmB) served as the positive control. After thorough mixing, 5 μL of the fungal suspension was spotted onto the SDA medium containing SDS and Congo Red, followed by incubation at 37 °C for 48 hours for observation and photography. Additionally, an alkaline phosphatase assay was conducted to determine whether the cell wall was compromised.

1.1.2 Effect of Isobavachalcone on the Biofilm of *Candida albicans*

The inhibitory effect of isobavachalcone on biofilm formation was evaluated using a modified crystal violet staining assay. Briefly, a sterile polypropylene 96-well plate was inoculated with 100 μL of *Candida albicans* suspension (1×10⁶ colony forming unit [CFU]/mL in Sabouraud Dextrose Broth, SDB) and incubated statically at 37 °C for 48 hours to allow biofilm formation. After aspirating the medium, fresh SDB containing isobavachalcone (4, 16, and 64 μg/mL) was added to each well, followed by incubation for an additional 24 hours.

The biofilms were gently washed twice with 100 μL phosphate-buffered saline (PBS), fixed with 100 μL methanol for 10 minutes, and then stained with 0.1% (w/v) crystal violet solution (Macklin, China) for 15 minutes. Excess stain was removed by repeated PBS washes until no visible color remained. Bound crystal violet was solubilized with 100 μL absolute ethanol, and the plates were mixed thoroughly on a horizontal shaker (60 rpm) at room temperature for 1 hour. Absorbance at 580 nm was measured using a microplate reader (BioTek Synergy H1, USA). Statistical analysis was performed using one-way analysis of variance.

1.1.3 Effect of Isobavachalcone on the Hydrophobicity of *Candida albicans*

The cell surface hydrophobicity (CSH) of *Candida albicans* was determined using a water-hydrocarbon two-phase assay. A fungal suspension of 1×10⁶ CFU/mL was added to a 6-well plate and co-cultured with different concentrations of isobavachalcone in a 37 °C incubator for 12 hours. The fungal cells were then centrifuged to harvest and resuspended in Yeast Extract-Peptone-Dextrose Medium medium, and the suspension was adjusted to an optical density (OD) of 1 at 600 nm (2). Subsequently, 1.2 mL of the adjusted fungal suspension was mixed with 0.3 mL of n-octane in a glass tube, vortex-mixed vigorously for 3 minutes, and allowed to stand for phase separation. The OD of the aqueous phase was immediately measured at 600 nm, and the hydrophobicity rate (CSH) was calculated using the formula: CSH (%) = (OD_600_sample / OD_600_control) × 100%.

1.1.4 Effect of Isobavachalcone on the Exopolysaccharides (EPS) of *Candida albicans*

The extracellular polysaccharides (EPS) were extracted following a modified protocol based on established methods in the literature. Briefly, a fungal suspension of *Candida albicans* (1×10⁶ CFU/mL) was incubated with varying concentrations of isobavachalcone for 6 hours. The culture was centrifuged to collect the supernatant, which was then mixed with absolute ethanol and kept at 4 overnight to precipitate EPS. The precipitate was harvested by centrifugation, air-dried at 37 °C, and dissolved in sterile distilled water to obtain the polysaccharide solution.

For quantification, 100 μL of the EPS solution was transferred to a sterile centrifuge tube and thoroughly mixed with 5% phenol. Concentrated sulfuric acid was slowly added using a pipette, followed by vigorous mixing. After cooling to room temperature for 30 minutes, the mixture was transferred to a 96-well plate, and the absorbance at 490 nm was measured using a microplate reader. The polysaccharide content was calculated based on a glucose standard curve (3).

1.2 Results

Fig. S1. Cell membrane integrity was assessed using 0.01% (w/v) sodium dodecyl sulfate (SDS) and 1 mg/mL Congo Red.

**
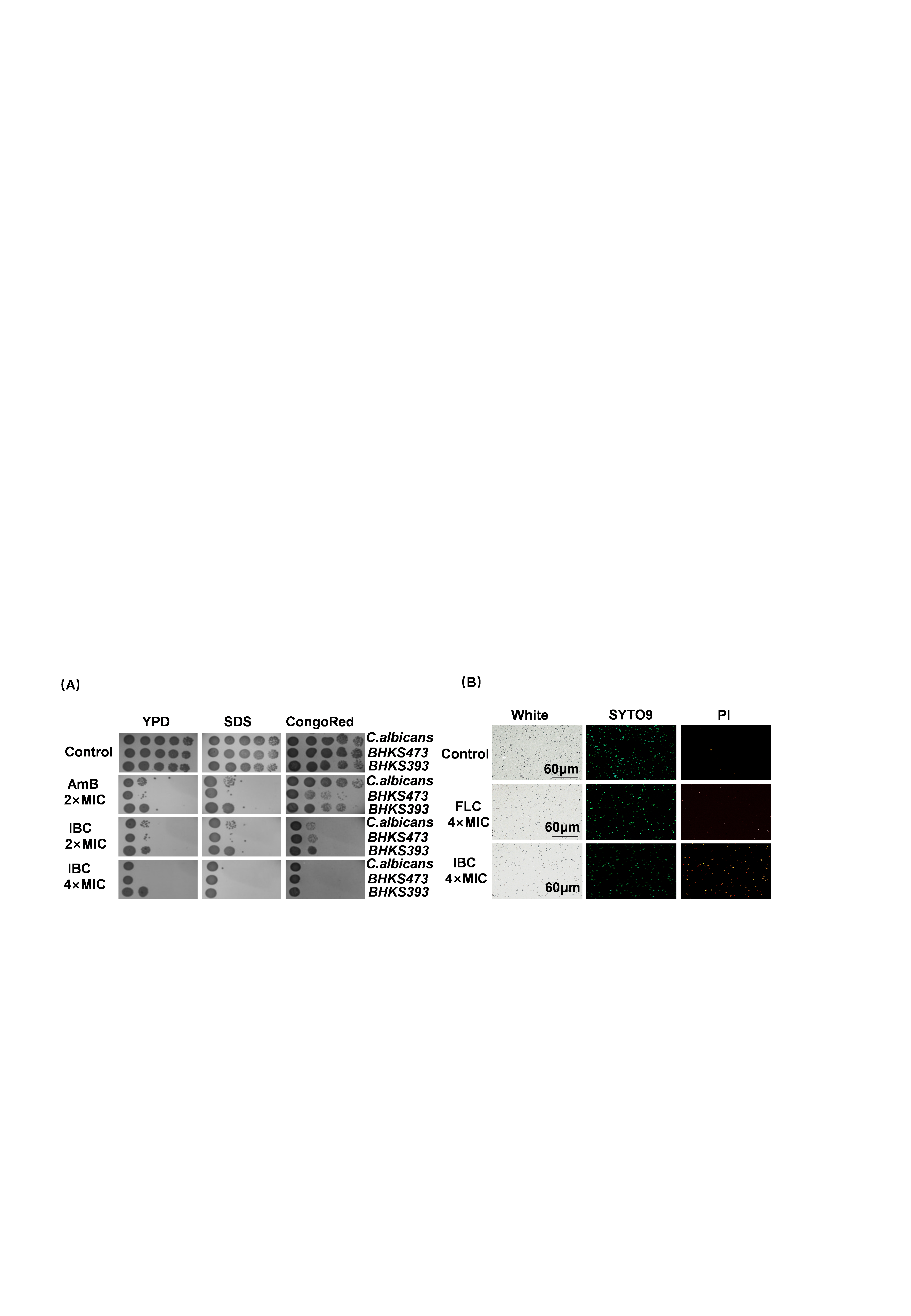
**

Fig. S2. (A) Effect of isobavachalcone on hyphal formation in spider medium，(B) Quantitative analysis of hyphal growth in spider medium. (C) Effect of isobavachalcone on biofilm formation. (D) Measurement of hydrophobicity in *Candida albicans* Treated with isobavachalcone. (E) Determination of exopolysaccharides (EPS) content in *Candida albicans* biofilm


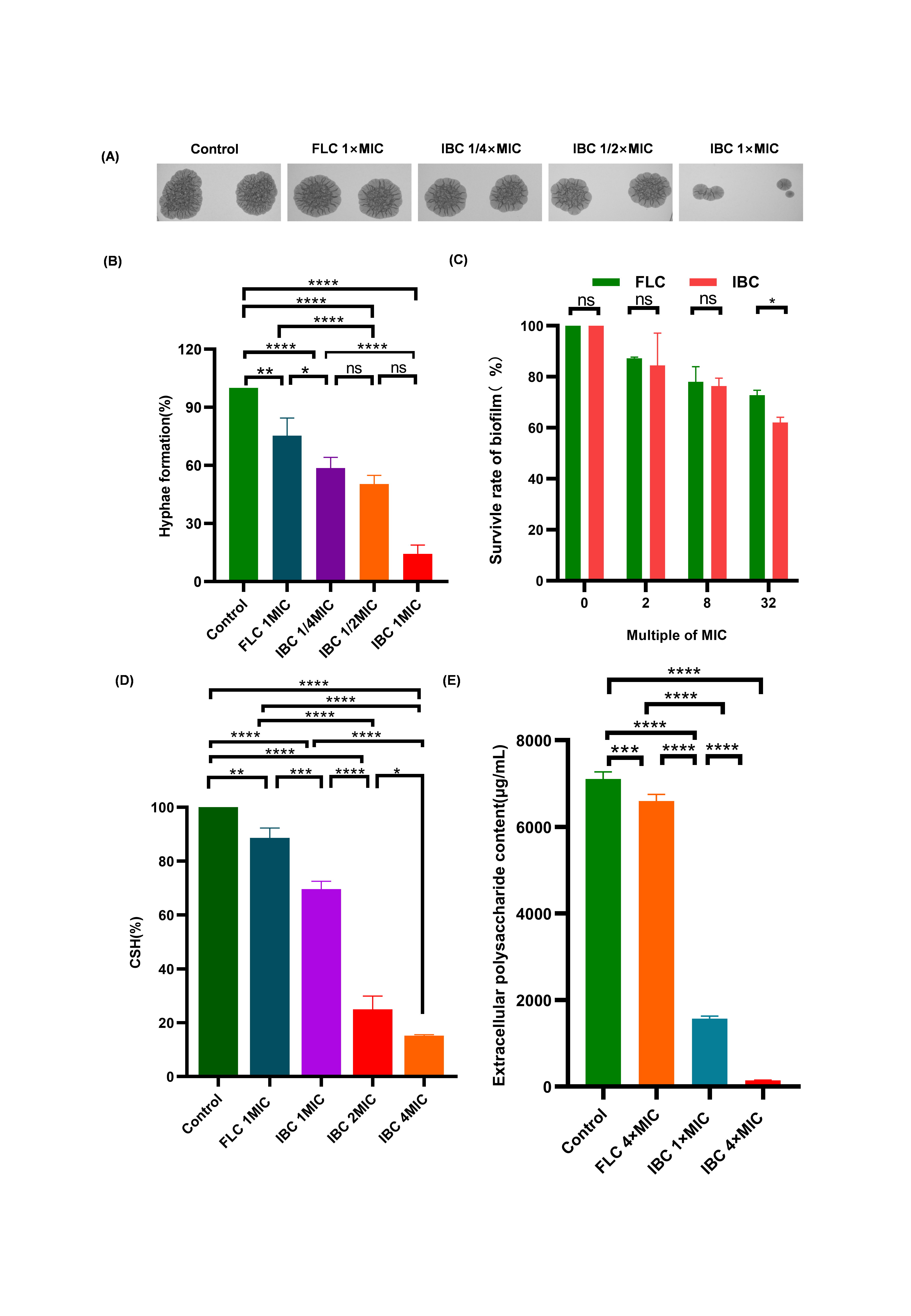


Fig S3 *Candida albicans* load in vaginal lavage fluid measured during isobavachalcone treatment of murine vaginitis induced by *Candida albicans.*


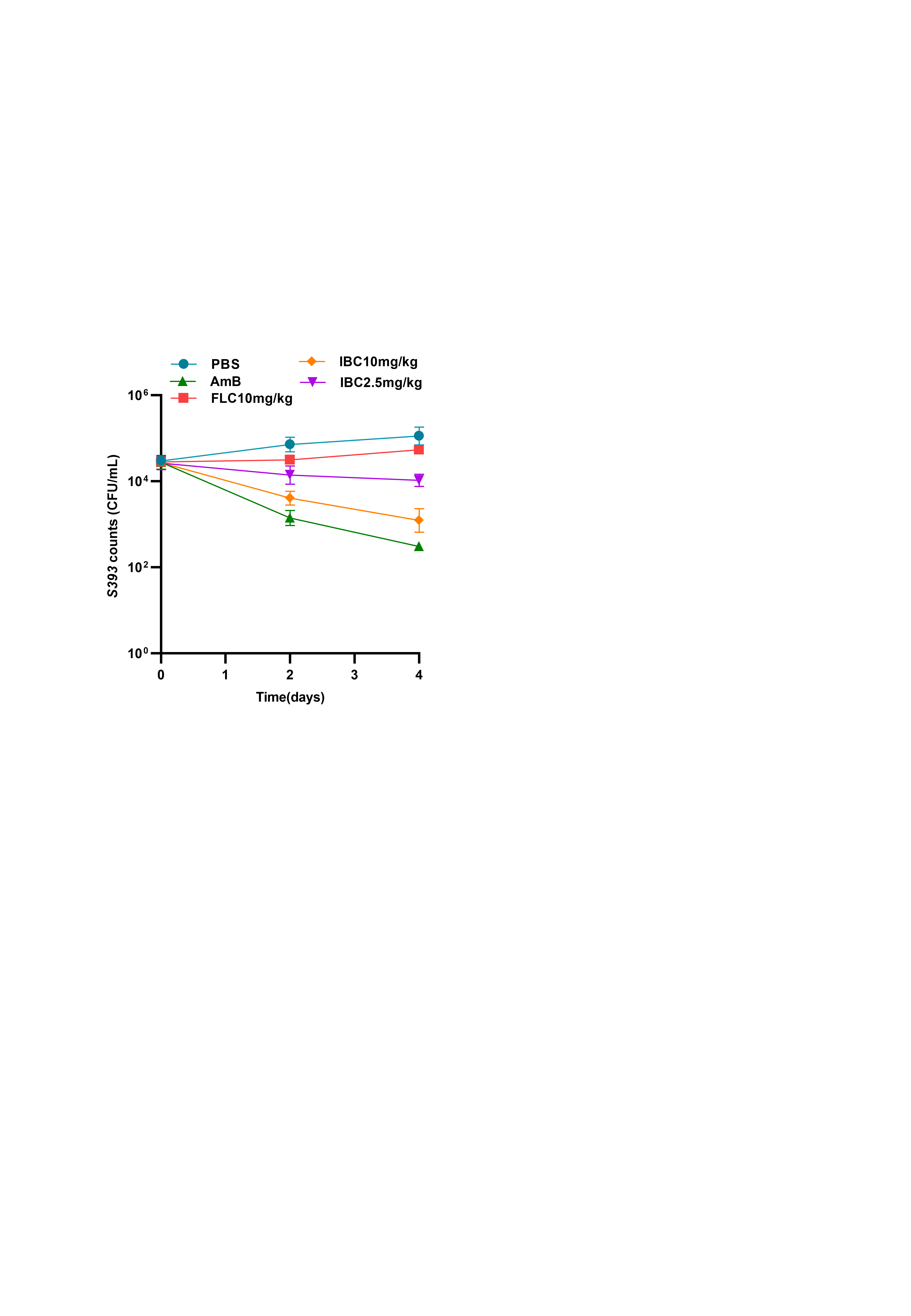


Fig S4 The PBS group, which was infected with oral thrush but received no treatment, exhibited the lowest survival rate at 33.3%. In comparison, the survival rates were 60% in the amphotericin B group and 75% in the isobavachalcone-treated group. Among these three groups, mice treated with isobavachalcone demonstrated the highest survival rate, indicating the therapeutic advantage of this compound.


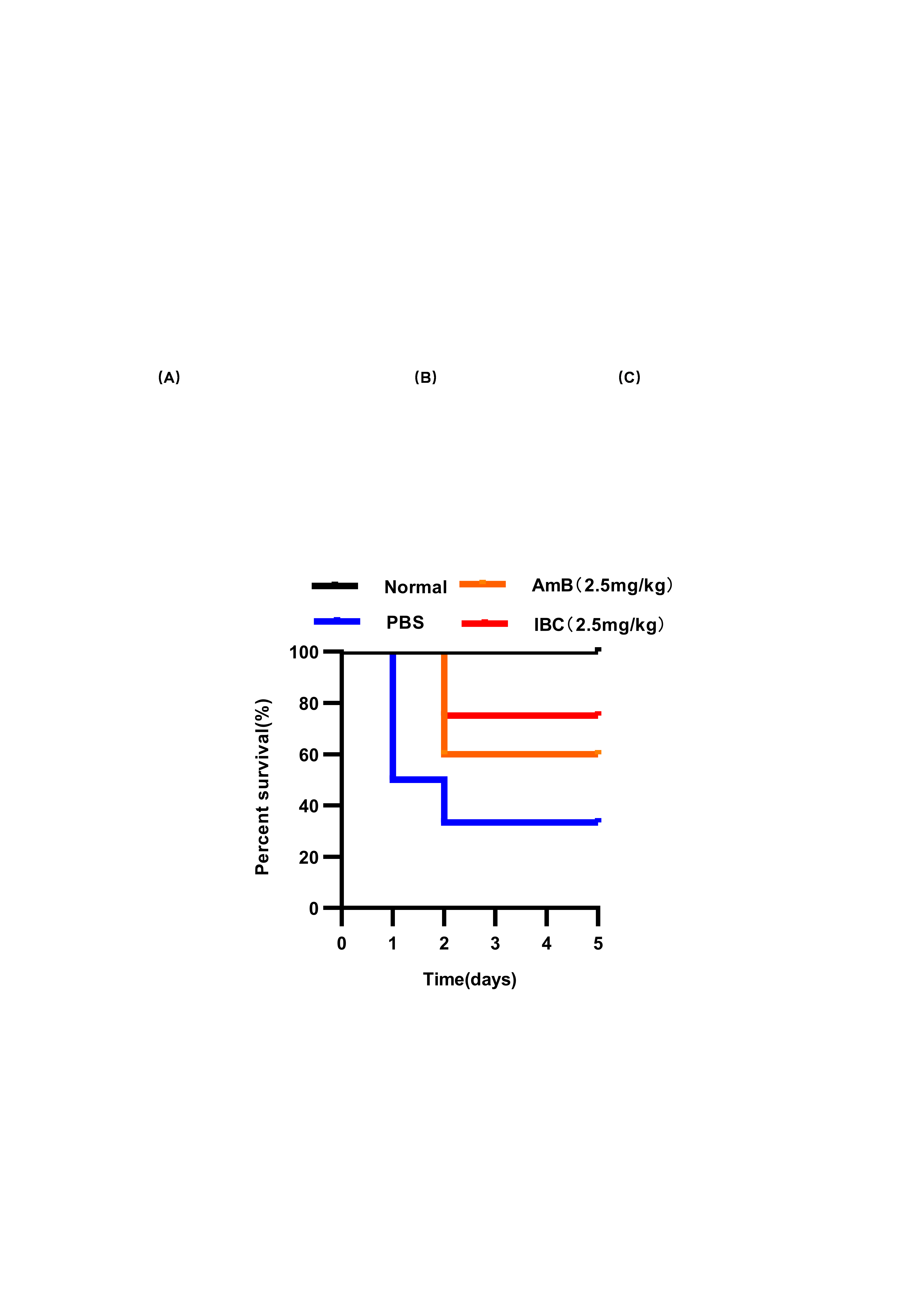


**REFERENCES**

1. Yockey J, Andres L, Carson M, et al. Cell Envelope Integrity and Capsule Characterization of Rhodotorula mucilaginosa Strains from Clinical and Environmental Sources. mSphere, 2019,4(3).doi: 10.1128/mSphere.00166-19.
2. Jiang Q, Jing Q, Ren B, Cheng L, Zhou X, Lai W, He J, Li M. Culture Supernatant of Enterococcus faecalis Promotes the Hyphal Morphogenesis and Biofilm Formation of *Candida albicans*. Pathogens. 2022 Oct ,11(10):1177.doi: 10.3390/pathogens11101177.
3. Liu Y, Wang Z, Zhou Z, Ma Q, Li J, Huang J, Lei L, Zhou X, Cheng L,Zou J, Ren B. *Candida albicans* CHK1 gene regulates its cross-kingdom interactions with Streptococcus mutans to promote caries. Appl Microbiol Biotechnol. 2022 ,106(21):7251-7263.doi: 10.1007/s00253-022-12211-7.

Table S1. Minimum Inhibitory Concentration (MIC_90_) of drugs against different *Candida* species (μg/mL)

| **No.** | **Strain Name** | **MIC_90_ (**μg/mL**)** | | | | | |
| --- | --- | --- | --- | --- | --- | --- | --- |
|  |  | FLC | KCZ | ITZ | 5-FC | AmB | IBC |
| 1 | *BHKS393* | 64 | 16 | >128 | >128 | 0.5 | 2 |
| 2 | *BHKS473* | >128 | 32 | >128 | >128 | 0.25 | 2 |
| 3 | *BHKS474* | >128 | 32 | >128 | >128 | 0.5 | 2 |
| 4 | *BHKS475* | >128 | 32 | >128 | >128 | 0.5 | 2 |
| 5 | *BHKS476* | >128 | 32 | >128 | >128 | 0.5 | 2 |
| 6 | *BHKS477* | >128 | 32 | >128 | >128 | 0.5 | 2 |
| 7 | *C. albicans* | >128 | 32 | >128 | >128 | 0.25 | 2 |

FLC: Fluconazole, KCZ: Ketoconazole, ITZ: Itraconazole, AmB: Amphotericin B, IBC: Isobavachalcone

Table S2. Primer list

| **Primer Designation** | **Forward primers 5’→3’** | **Reverse primers 5’→3’** |
| --- | --- | --- |
| *ITS* | TGCCTGTTTGAGCGTCGTTTC | CGATCCCGCCTTACCACTACC |
| *ADK1* | GGTTTTGATCGGTCCTCCAGGTG | AAAGCGGTTTTAGCAGCAACTTGTG |
| *ADE13* | GTGAAACTTGTCCTCTGGCTGCTG | ACTGTGGTCAATTGTGCTGGTTGG |
| *TPI1* | GTTTAGCCGCTACCCCAGAAGATG | ACCGTTGACTGAACCACCATACAAG |
| *ADH2* | TGGTGTTGTTGTCGCCTTGGG | CTTCAGCACAGTTTGGTTCAGCAC |

**Table S3** Predicted binding energies for docking of IBC to target proteins

| **Accession** | **Gene** | **Mw (kDa)** | **Docking_score (kcal/mol)** |
| --- | --- | --- | --- |
| Q5A4Q1 | *ADK1* | 27.58 | -7.5 |
| A0A1D8PT56 | *ADE13* | 29.578 | -7.1 |
| Q9P940 | *TPI1* | 26.611 | -6.6 |
